# Supplementary material for: Cell size and actin architecture determine force generation in optogenetically activated cells
Source: Biophys J. 2023 Jan 12;122(4):684–96. doi: 10.1016/j.bpj.2023.01.011 (PMC9989885; doi:10.1016/j.bpj.2023.01.011)
Supplement: Document S1. Figures S1–S5 and Tables S1–S4 [file mmc1.pdf]

Biophysical Journal, Volume *122*

Supplemental information

Cell size and actin architecture determine force generation in optogenetically activated cells

T. Andersen, D. Wörthmüller, D. Probst, I. Wang, P. Moreau, V. Fitzpatrick, T. Boudou, U.S. Schwarz, and M. Balland

Cell size and actin architecture determine force generation in optogenetically activated cells

T. Andersen, D. Wörthmüller, D. Probst, I. Wang, P. Moreau, V. Fitzpatrick, T. Boudou, U.S. Schwarz and M. Balland

Supplemental Figure S1: Cells with similar actin organization display identical force response independently of the pattern adhesive area

(a) Substrate displacement measured with respect to distance from the cell edge along the lines in Fig. 1b. Dotted lines correspond to exponential fits according to the theoretical decay for the displacements. The corresponding values for l_p are 5.6 μm (500 μm^2), 8.7 μm (1000 μm^2) and 12.0 μm (1500 μm^2). (b) Comparison of the three different measures for displacement decay as a function of cell size: half-maximum values from Fig. 1c, exponential fits from Fig. S1a, model-fits to baseline from Fig. 2c. They all show similar values and the same increasing trend with cell size. (c) From left to right: 1000 μm^2 disc, donut and hazard shaped fibronectin micropatterns on polyacrylamide (all patterns cover the same projected area). Individual actin-labelled cells. Individual vinculin staining to reveal focal adhesion localization. The contrast of the vinculin images is enhanced to facilitate visualization of small and thin focal adhesions. (d) Total adhesion area measured as integrated vinculin signal on the 1000 μm^2 disc, donut and hazard shapes. Using a 1-way ANOVA test, significant difference is not found between the three cases. (e) Global cellular actin fibre alignment for cells spread on all fibronectin micropatterns. This is represented by the actin order parameter. Using a 1-way ANOVA test, no significant difference is found between the disc and the donut, however, the hazard pattern displays significant differences with both patterns. (f) Normalized quantification of the mean strain energy over time for cells on all shapes subjected to one light pulse of 100 ms. (g) Strain energy increase for every activated cell on the three different shapes. Calculation is made by subtracting the strain energy value before activation to the highest strain energy value obtained after light activation. Only cells plated on the hazard shaped micropattern displayed lower efficiency in terms of strain energy increase after photoactivation.

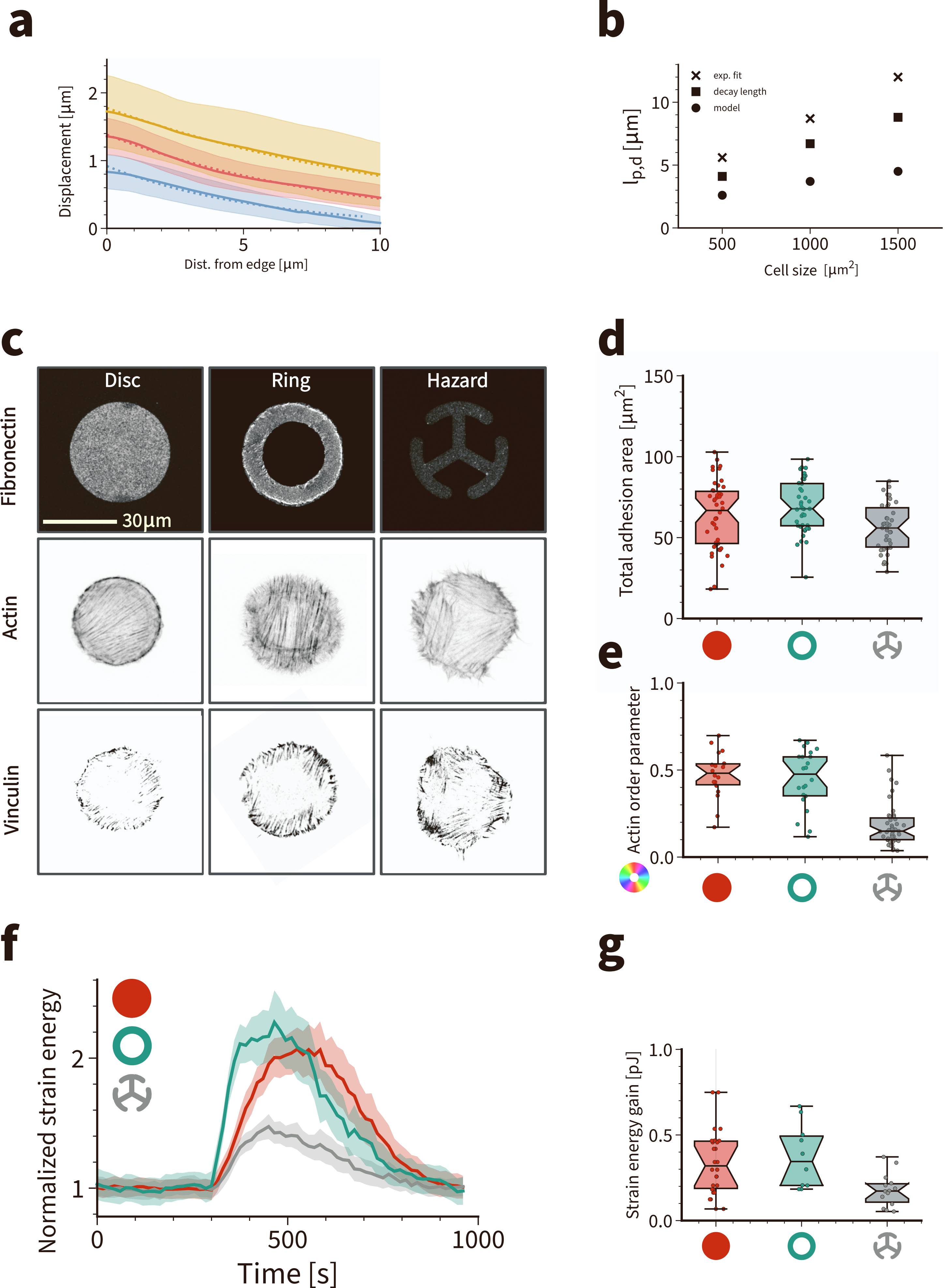

a
Displacement [µm]
Dist. from edge [µm]
b
exp. fit
decay length
model
l_p,d [µm]
Cell size [µm²]
c
Disc
Ring
Hazard
Fibronectin
Actin
Vinculin
30µm
d
Total adhesion area [µm²]
e
Actin order parameter
f
Normalized strain energy
Time [s]
g
Strain energy gain [pJ]

Cell size and actin architecture determine force generation in optogenetically activated cells
# - Supplemental text -

T. Andersen, D. Wörthmüller, D. Probst, I. Wang, P. Moreau, V. Fitzpatrick, T. Boudou, U.S. Schwarz and M. Balland

Contents

1 Overview 2

2 Mechanical model 2

3 Substrate strain energy 3

4 Analytical solution for contractile disc 4

5 Numerical implementation 6

6 Adhesive geometry 7

7 Photoactivation 9

8 Parametrization 11

9 Scaling considerations from analytical model 12

10 Effect of actin organization on strain energy level in the model 13

11 Example for python FEM-code for contractile disc 14

1 Overview

Our modelling approach has to combine the following elements: it has to represent the geometry of the adhesive environment of the cell, it has to describe the active mechanical properties of the cell, in particular the effect of photoactivation, and it has to predict the strain energy of the elastic substrate, which shows a characteristic time course after photoactivation. Here we introduce a modelling framework that meets all of these requirements. Our presentation is structured as follows. We first introduce our mechanical model and briefly discuss potential alternatives that we also tested. We then discuss the analytical solutions that are possible for the special assumption of isotropic contractility on a disc pattern. These analytical solution are used to validate our numerical treatment and are helpful to parametrize our model. Next we discuss the numerical implementation of your model, which is needed for the general case of anisotropic contractility. This section also includes a description of how we implement the different adhesive geometries. We then discuss photoactivation, in particular our choice of a double-sigmoid activation curve and potential alternatives. Finally we summarize our parametrization of the model. Here we adopt a mixed strategy. One subset of the parameters is taken from general considerations, and the complementary part is determined by numerical minimizing the loss against experimental data. All parameter values used for the calculations are documented in tables.

2 Mechanical model

We start with the mechanical model. Following earlier work on modelling traction forces as a function of cell geometry [1, 2, 3, 4, 5, 6, 7, 8], we describe the cell as a thin contractile layer that adheres to an elastic foundation (compare Fig. 2a). The force balance between the cell and the substrate reads

$$\partial_j \sigma_{ij}(\mathbf{x}, t) = Y(\mathbf{x}) u_i(\mathbf{x}, t)\,. \tag{1}$$

Here σ_{ij} is the two-dimensional stress in the contractile layer, u_i its displacement field and Y the local area density of the spring constants. Y therefore represents the stiffness of the foundation. Through its position dependance, $Y(\mathbf{x})$ can also represent the adhesive geometry.

For the constitutive law of the contractile layer we choose an active Kelvin-Voigt model:

$$\sigma_{ij} - \sigma_{ij,\mathrm{m}} = (1 + \tau_\mathrm{c} \partial_t)\left(\lambda \epsilon_{kk} \delta_{ij} + 2\mu \epsilon_{ij}\right) \tag{2}$$

with linear strain tensor $\epsilon_{ij} = (\partial_i u_j + \partial_j u_i)/2$. σ_m denotes the active motor stress, which consists of two contributions: a constant background stress σ_bck, which raises the cellular strain energy to its homeostatic level, and a photoactivation (PA) stress tensor $\sigma_\mathrm{act}(t)$, describing the additional time-dependent stress during PA. λ and μ denote the two-dimensional Lamé coefficients defined by

$$\lambda = \frac{E_\mathrm{c} h_\mathrm{c} \nu_\mathrm{c}}{1 - \nu_\mathrm{c}^2}\,, \quad \mu = \frac{E_\mathrm{c} h_\mathrm{c}}{2\left(1 + \nu_\mathrm{c}\right)}\,. \tag{3}$$

Here E_c and ν_c are the three-dimensional Young's modulus and Poisson's ratio of the cells, respectively, and h_c is the effective thickness of the contractile layer, which is similar to but smaller than cell

thickness. The effective viscosity of the cell will be denoted by η_{c} and the resulting relaxation time is $\tau_{\mathrm{c}} = \eta_{\mathrm{c}}/E_{\mathrm{c}}$. The limit $\tau_{\mathrm{c}} = 0$ corresponds to the purely elastic case. Because elastic and viscous elements are arranged in parallel in the Kelvin-Voigt model, the corresponding forces simply add up in this equation.

An alternative to the active viscoelastic solid is the active viscoelastic fluid, that is an active Maxwell model, for which we have

$$\frac{\sigma_{ij} - \sigma_{ij,\mathrm{m}}}{\tau_{\mathrm{c}}} + \left(\dot{\sigma}_{ij} - \dot{\sigma}_{ij,\mathrm{m}}\right) = \partial_t \left(\lambda \epsilon_{kk} \delta_{ij} + 2\mu \epsilon_{ij}\right) \; . \tag{4}$$

$\tau_{\mathrm{c}} = 0$ corresponds to the purely viscous case, for which the stress derivative would vanish. Because now elastic and viscous elements for the viscoelastic fluid are arranged in series, they appear here in a different combination than for the viscoelastic solid from Eq. 2. For this study, we considered all four possible linear models (viscoelastic solid, elastic solid, viscoelastic fluid, viscous fluid), but only the viscoelastic solid was able to describe our experimental data (see below).

In order to solve our model, we have to combine the force balance from Eq. 1 with the constitutive law from Eq. 2. In general, the resulting equation can only be solved numerically. If one considers the special case of a one-dimensional and purely elastic system ($\tau_{\mathrm{c}} = 0$) with constant stiffness Y and constant active stress σ^{m}, the resulting equation for the displacement field u is simply

$$\partial_x^2 u - \frac{1}{l_p^2} u = 0 \tag{5}$$

with the newly defined force penetration length l_{p} and the stiffness Y related by

$$l_p = \left(\frac{E_{\mathrm{c}} h_{\mathrm{c}}}{Y\left(1 - \nu_{\mathrm{c}}^2\right)}\right)^{1/2} \quad , \quad Y = \frac{E_{\mathrm{c}} h_{\mathrm{c}}}{l_{\mathrm{p}}^2 \left(1 - \nu_{\mathrm{c}}^2\right)} \; . \tag{6}$$

The force penetration length can be understood as the typical length scale on which a mechanical perturbation decays [1]. In principle this allows us to estimate Y by experimentally measuring l_{p}.

3 Substrate strain energy

In order to predict the substrate strain energy in our model, we take into account that both the elastic gel and the layer of adhesion molecules connecting it to the cell contribute to the foundation stiffness Y perceived by the cell. Because the stiffnesses of the substrate and of the adhesion layer act in series, we write $1/Y = 1/Y_{\mathrm{s}} + 1/Y_{\mathrm{a}}$. The spring constant density of the substrate is related to its Young's modulus E_{s} by [2]

$$Y_{\mathrm{s}} = \frac{\pi E_{\mathrm{s}}}{h_{\mathrm{eff}}} \tag{7}$$

where h_{eff} can be estimated as

$$h_{\mathrm{eff}}^{-1} = \frac{1}{h_{\mathrm{s}} 2\pi \left(1 + \nu_{\mathrm{s}}\right)} + \frac{1}{L_{\mathrm{c}}} \tag{8}$$

where h_s and ν_s are thickness and Poisson ratio of the substrate, respectively, and L_c is the lateral size of the cell. The stiffness of the adhesion layer can be estimated as $Y_\mathrm{a} = k_a/d^2$, where k_a is the molecular stiffness of the adhesion bonds and d the distance between them.

With substrate displacement $\mathbf{u}_\mathrm{s}$, substrate rigidity Y_s and force balance $\mathbf{T} = Y\mathbf{u} = Y_\mathrm{s}\mathbf{u}_\mathrm{s}$, we now can write for the substrate strain energy:

$$U_\mathrm{s} = \frac{1}{2}\int_A \mathbf{T}\mathbf{u}_\mathrm{s}\mathrm{d}A = \frac{1}{2}\int_A \frac{Y^2}{Y_\mathrm{s}}\mathbf{u}^2\mathrm{d}A \ . \tag{9}$$

This is the central quantity of interest because it is directly measured in the experiments. If the adhesion layer is much stiffer than the elastic substrate, we have $Y = Y_\mathrm{s}$ and the energy density is simply $Y_\mathrm{s}\mathbf{u}^2/2$.

4 Analytical solution for contractile disc

To estimate the expected values for the strain energy U_s described by Eq. 9 as well as the contractile background stress of the cell, we now turn to an analytical solution of our model that has been derived before for the case of an isotropic contractile disc of radius r_0 [1, 4]. Here we restrict ourselves completely to mechanical equilibrium and neglect viscoelastic effects or non-homogeneous adhesion. The radial displacement u_r for this special case yields [1]

$$u_r(r) = -l_\mathrm{p}\frac{\sigma_0 h_\mathrm{c}}{\lambda + 2\mu}\cdot\frac{I_1\left(\frac{r}{l_\mathrm{p}}\right)}{I_0\left(\frac{r_0}{l_\mathrm{p}}\right) - \frac{2\mu}{\lambda+2\mu}\frac{l_\mathrm{p}}{r_0}I_1\left(\frac{r_0}{l_\mathrm{p}}\right)} \ , \tag{10}$$

with contractile stress σ_0, disc height h_c and modified Bessel functions of first kind I_0 and I_1. The strain energy then reduces to the integral

$$\begin{aligned} U_\mathrm{s} &= \frac{Y^2}{2Y_\mathrm{s}}\int_0^{2\pi}\mathrm{d}\phi\int_0^{r_0}\mathrm{d}r r u_r^2 \\ &= \frac{\pi}{Y_\mathrm{s}}\cdot\left(\frac{Y l_\mathrm{p}\sigma_0 h_\mathrm{c}\left(1-\nu_\mathrm{c}^2\right)}{E_\mathrm{c}h_\mathrm{c}}\right)^2\cdot\frac{\int_0^{r_0}\mathrm{d}r r I_1\left(\frac{r}{l_\mathrm{p}}\right)^2}{\left(I_0\left(\frac{r_0}{l_\mathrm{p}}\right) - \left(1-\nu_\mathrm{c}\right)\frac{l_\mathrm{p}}{r_0}I_1\left(\frac{r_0}{l_\mathrm{p}}\right)\right)^2} = \frac{\pi\left(\sigma_0 h_\mathrm{c}\right)^2}{2Y_\mathrm{s}}\zeta\left(\frac{r_0}{l_\mathrm{p}}\right) \ , \end{aligned} \tag{11}$$

using the definition of Y in Eq. 6 and

$$\zeta(x) = x^2\cdot\frac{I_1(x)^2 + \frac{2}{x}I_0(x)I_1(x) - I_0(x)^2}{\left(I_0(x) - \left(1-\nu_\mathrm{c}\right)\frac{1}{x}I_1(x)\right)^2} \ . \tag{12}$$

The total traction force exerted onto the substrate is given by

$$\begin{aligned} F_\mathsf{tot} &= Y\int_0^{2\pi}\mathrm{d}\phi\int_0^{r_0}\mathrm{d}r r|u_r| \\ &= \frac{2\pi Y l_p\sigma_0 h_c}{\lambda + 2\mu}\frac{\int_0^{r_0}\mathrm{d}r r I_1\left(\frac{r}{l_p}\right)}{I_0\left(\frac{r_0}{l_\mathrm{p}}\right) - \left(1-\nu_\mathrm{c}\right)\frac{l_\mathrm{p}}{r_0}I_1\left(\frac{r_0}{l_\mathrm{p}}\right)} = \frac{2\pi\sigma_0 h_c}{l_p}\beta\left(\frac{r_0}{l_p}\right) \ , \end{aligned} \tag{13}$$

with

$$\beta\left(x\right)=\frac{\pi}{2}x\frac{\left(L_{0}(x)I_{1}(x)-L_{1}(x)I_{0}(x)\right)}{I_{0}\left(x\right)-\left(1-\nu_{c}\right)\frac{1}{x}I_{1}\left(x\right)}\;, \tag{14}$$

in which L_0 and L_1 denote modified Struve functions.

We can find the two asymptotic limits of the strain energy and the total traction force by investigating ζ and β for the two limits $x \ll 1$ and $x \gg 1$. For $x \ll 1$, the modified Bessel functions can be approximated as

$$I_{n}\left(x\right)\xrightarrow[x\ll 1]{}\frac{1}{n!}\left(\frac{x}{2}\right)^{n}\;, \tag{15}$$

such that

$$\zeta\left(x\right)\xrightarrow[x\ll 1]{}\frac{x^{4}}{2\left(1+\nu_{\mathrm{c}}\right)^{2}}+\mathcal{O}\left(x^{5}\right)\;, \tag{16}$$

$$\beta\left(x\right)\xrightarrow[x\ll 1]{}\frac{x^{3}}{3\left(1+\nu_{\mathrm{c}}\right)}+\mathcal{O}\left(x^{4}\right)\;, \tag{17}$$

and hence

$$U_{\mathrm{s}}\xrightarrow[r_0\ll l_{\mathrm{p}}]{}\frac{\pi\left(\sigma_{0}h_{\mathrm{c}}\right)^{2}}{4Y_{\mathrm{s}}\left(1+\nu_{\mathrm{c}}\right)^{2}}\left(\frac{r_{0}}{l_{\mathrm{p}}}\right)^{4}\;, \tag{18}$$

$$F_{\mathsf{tot}}\xrightarrow[r_0\ll l_{\mathrm{p}}]{}\frac{2\pi l_{p}\sigma_{0}h_{c}}{3(1+\nu_{c})}\left(\frac{r_{0}}{l_{p}}\right)^{3}\;. \tag{19}$$

For $x \gg 1$, any modified Bessel function of the first kind can be approximated as

$$I_{n}\approx\frac{\exp x}{\sqrt{2\pi x}}\left[1-\frac{4n^{2}-1^{2}}{1(8x)}\left(1-\frac{4n^{2}-3^{2}}{2(8x)}\left(1-\frac{4n^{2}-5^{2}}{3(8x)}\left(1-\ldots\right)\right)\right)\right]\;, \tag{20}$$

i.e.

$$I_{0}\approx\frac{\exp x}{\sqrt{2\pi x}}\left[1+\frac{1}{8x}\right] \tag{21}$$

$$I_{1}\approx\frac{\exp x}{\sqrt{2\pi x}}\left[1-\frac{3}{8x}\right] \tag{22}$$

such that

$$\zeta\left(x\right)\approx x\frac{64-\frac{24}{x}-\frac{6}{x^{2}}}{64-\frac{48}{x}+\frac{3}{x^{2}}-\frac{9}{x^{3}}+\frac{9}{4x^{4}}}\xrightarrow[x\gg 1]{}x+\mathcal{O}\left(x^{2}\right)\;, \tag{23}$$

$$\beta\left(x\right)\approx x\frac{1-\frac{7}{8x}}{1+\frac{1}{8x}-\left(1-\nu_{c}\right)\frac{1}{x}-\frac{3}{8x^{2}}}\xrightarrow[x\gg 1]{}x+\mathcal{O}\left(x^{2}\right)\;, \tag{24}$$

and hence

$$U_{\mathrm{s}}\xrightarrow[r_0\gg l_{\mathrm{p}}]{}\frac{\pi\left(\sigma_{0}h_{\mathrm{c}}\right)^{2}}{2Y_{\mathrm{s}}}\cdot\frac{r_{0}}{l_{\mathrm{p}}}\;, \tag{25}$$

$$F_{\mathsf{tot}}\xrightarrow[r_0\gg l_{\mathrm{p}}]{}2\pi\sigma_{0}h_{c}r_{0}\;. \tag{26}$$

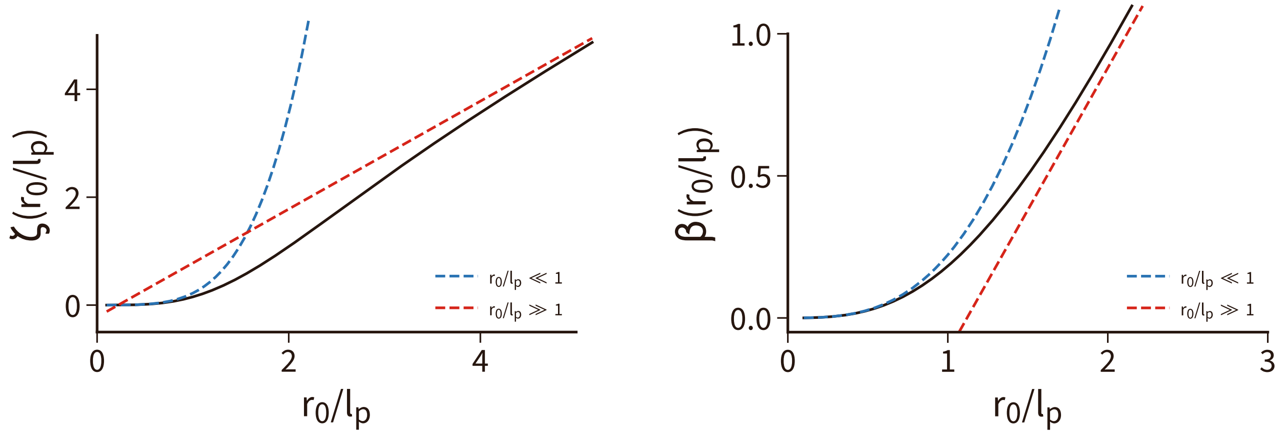

Figure 1: Analytical solution for the substrate strain energy U_s and the total traction force F_{tot} of a uniformly contractile disc as a function of dimensionless disc radius r_0/l_p. Also shown are the two asymptotic limits for small (blue) and large (red) disc radii. For radii r_0 larger than l_p, a linear approximation becomes valid in both cases.

Thus, the asymptotic behavior of the strain energy for $r_0 \gg l_{\rm p}$ has the same scaling with r_0 as the total force, corresponding to a tension-dominated regime. However, for $r_0 \ll l_{\rm p}$, the asymptotic behavior of the strain energy and the total force have a different scaling with r_0, corresponding to an elasticity-dominated regime. As we will see below, in our experiments we always will deal with the tension-dominated regime.

In Fig. 1, we plot the substrate strain energy U_s from Eq. 11 and total traction force F_{tot} from Eq. 13 of the isotropically contracting disc as a function of dimensionless disc radius r_0/l_p (black solid line). We also plot the asymptotic limits for small radius (blue dashed line, Eq. 18) and large radius (red dashed line, Eq. 25). Because in experiments system size will be typically much larger than l_p, we conclude that the linear scaling from the large radius limit best captures the relevant form of the substrate strain energy and total traction force.

5 Numerical implementation

In the general case, no analytical solution is available and our mechanical model has to be solved by means of a finite element (FE) calculation. The weak formulation of Eq. 1 is

$$\int_\Omega \boldsymbol{\sigma} : \frac{1}{2}\left(\nabla\mathbf{v} + \nabla\mathbf{v}^{\mathbf{T}}\right)\mathrm{d}\mathbf{x} + \int_\Omega Y\,\mathbf{u}\cdot\mathbf{v}\,\mathrm{d}\mathbf{x} = 0\,, \tag{27}$$

with Ω denoting the meshed cell area (e.g. a disc) and $\mathbf{v}$ a test function. We use the FE-solver FEniCS to calculate the displacements [9]. For symmetry reasons, the Dirichlet boundary condition $\mathbf{u} = (0,0)$ applies at the midpoint $\mathbf{x} = (0,0)$.

As a validation of our numerical procedures, we first simulated the contractile disc. Fig. 2 shows that the analytical solution from Eq. 10 and the numerical solution agree very well.

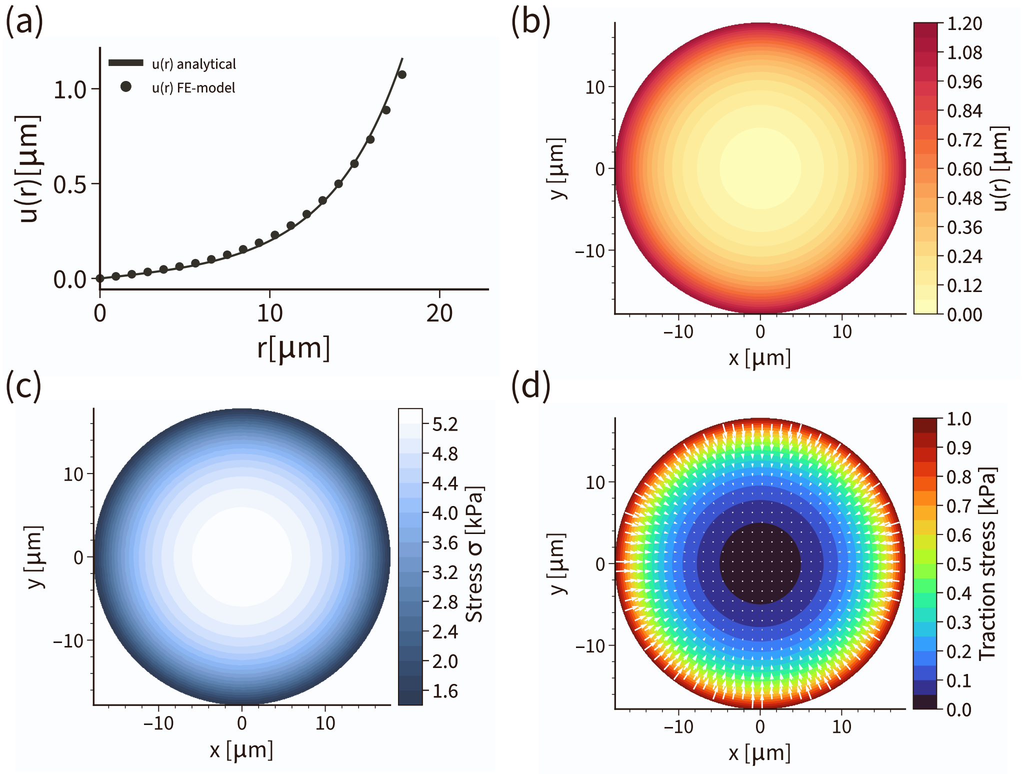

Figure 2: Finite Element Simulation of the isotropic contractile disc. Panel (a) demonstrates the excellent agreement of the numerical FE-implementation with the analytical solution for $u(r)$. Panel (b),(c) and (d) show the displacement field u, total stress σ and the traction stress as obtained by FEM, respectively.

6 Adhesive geometry

An essential element of our treatment is the representation of the adhesive geometry. This can be done by making stiffness Y space-dependent. For example, it has been shown recently that the arrangement of focal adhesions, and thus adhesion geometry, affects the effective substrate stiffness perceived by the cell [8]. For the case of the disc pattern, we represent the effect of the elastic substrate as well as the elastic contribution of the FAs via springs of constant spring stiffness density Y throughout the entire disc. For the hazard pattern, we only introduce springs of stiffness density Y at those positions of the disc at which the cell can form connections to the substrate via its FAs, which is exactly the FN coated area (illustrated in Fig. 3). To simulate this fact, we determine the positions $(x, y)_{Y\neq 0}$, at

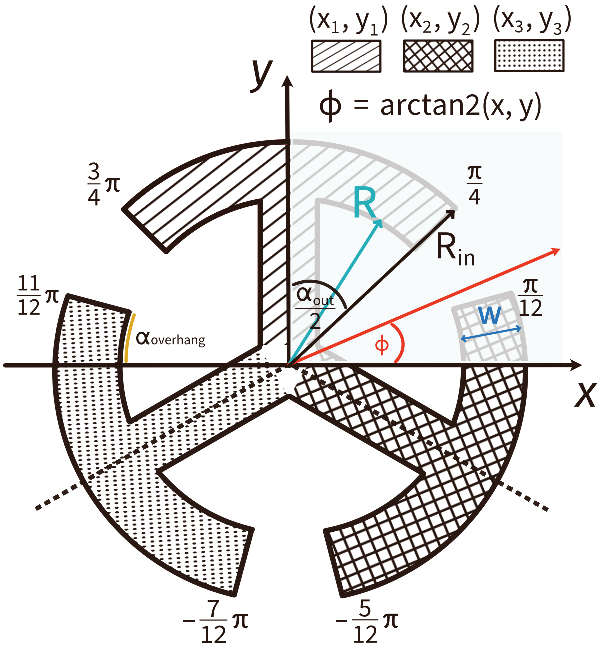

Figure 3: Illustration of the hazard pattern geometry. The relevant angles are described in the main text and implemented in the FEM-simulations.

which the stiffness density Y is non-zero, via:

$$
\begin{aligned}
(x,y)_{Y\neq 0} = \Bigg\{ x, y \Bigg| \\
& \Bigg[R_{\text{in}} \leq \sqrt{x^2+y^2} \leq R \wedge \Bigg(\frac{\pi}{2} - \frac{\alpha_{\text{out}}}{2} \leq \text{arctan2}\,(x,y) \leq \frac{\pi}{2} + \frac{\alpha_{\text{out}}}{2} \\
& \vee -\frac{\pi}{6} - \frac{\alpha_{\text{out}}}{2} \leq \text{arctan2}\,(x,y) \leq -\frac{\pi}{6} + \frac{\alpha_{\text{out}}}{2} \\
& \vee -\frac{5\pi}{6} - \frac{\alpha_{\text{out}}}{2} \leq \text{arctan2}\,(x,y) \leq -\frac{5\pi}{6} + \frac{\alpha_{\text{out}}}{2} \\
& \vee \pi - \alpha_{\text{overhang}} \leq \text{arctan2}\,(x,y) \leq \pi \Bigg) \Bigg] \vee \\
& \Bigg[\sqrt{x^2+y^2} \leq R_{\text{in}} \wedge \Bigg(\Bigg(-\frac{w}{2} \leq x_1 \leq \frac{w}{2} \ \wedge\ y_1 \geq 0 \Bigg) \\
& \vee \Bigg(-\frac{w}{2} \leq x_2 \leq \frac{w}{2} \ \wedge\ y_2 \geq 0 \Bigg) \\
& \vee \Bigg(-\frac{w}{2} \leq x_3 \leq \frac{w}{2} \ \wedge\ y_3 \geq 0 \Bigg) \Bigg) \Bigg] \Bigg\},
\end{aligned}
$$

with arm width $w = 5\,\mu\text{m}$, inner radius $R_{\text{in}} = R - w$ and $\alpha_{\text{out}} = {}^{\pi}/_{2}$. The remaining parameters are

$$
\begin{aligned}
(x_1, y_1) &= (x,\ y) \\
(x_2, y_2) &= \left(x \cdot \cos\left(\frac{2\pi}{3}\right) - y \cdot \sin\left(\frac{2\pi}{3}\right),\ x \cdot \sin\left(\frac{2\pi}{3}\right) + y \cdot \cos\left(\frac{2\pi}{3}\right) \right) \\
(x_3, y_3) &= \left(x \cdot \cos\left(\frac{2\pi}{3}\right) + y \cdot \sin\left(\frac{2\pi}{3}\right),\ -x \cdot \sin\left(\frac{2\pi}{3}\right) + y \cdot \cos\left(\frac{2\pi}{3}\right) \right),
\end{aligned}
$$

and

$$\alpha_{\text{overhang}} = \frac{5\pi}{6} + \frac{\alpha_{\text{out}}}{2} - \pi \text{ if } \frac{5\pi}{6} + \frac{\alpha_{\text{out}}}{2} > \pi, \text{ otherwise } 0\,. \tag{28}$$

Eq. 28 accounts for the unsteady jump of the $\arctan2$-function at the function values $-\pi$ and π.

7 Photoactivation

As explained above, the active stress has two components, $\sigma_{\text{m}} = \sigma_{\text{bck}} + \sigma_{\text{act}}(t)$, namely a background stress before PA, and the active stress after PA. 1 We assume that they both pull in the same direction because PA leads to little changes in the cytoskeleton, so the direction of pulling is not changed, but its strength is. We can calculate the anisotropic motor stress tensor σ_{m} directed along an arbitrary angle ϕ with respect to the x-axis via rotation of a stress tensor with its only non-zero component being $\sigma_{xx} = \sigma_{\text{bck}} + \sigma_{\text{act}}(t)$. Here, σ_{bck} is the background stress and σ_{act} is the time-dependent PA stress. One has

$$\begin{aligned}\sigma_{\text{m}}(\phi) &= \begin{pmatrix}\cos\phi & -\sin\phi \\ \sin\phi & \cos\phi\end{pmatrix}\begin{pmatrix}\sigma_{\text{bck}}+\sigma_{\text{act}} & 0 \\ 0 & 0\end{pmatrix}\begin{pmatrix}\cos\phi & \sin\phi \\ -\sin\phi & \cos\phi\end{pmatrix} \\ &= (\sigma_{\text{bck}}+\sigma_{\text{act}})\cdot\begin{pmatrix}\cos^2\phi & \frac{1}{2}\sin(2\phi) \\ \frac{1}{2}\sin(2\phi) & \sin^2\phi\end{pmatrix},\end{aligned} \tag{29}$$

Comparison with the orientation of SFs in cells plated on the two patterns lets us assume a motor stress tensor

$$\sigma_{\text{m}}^{\text{DP}} = \begin{pmatrix}0 & 0 \\ 0 & \sigma_{\text{bck}}+\sigma_{\text{act}}\end{pmatrix} \tag{30}$$

in the case of the disc pattern (DP) and

$$\begin{aligned}\sigma_{\text{m},\Omega1}^{\text{HP}} &= (\sigma_{\text{bck}}+\sigma_{\text{act}})\cdot\begin{pmatrix}1 & 0 \\ 0 & 0\end{pmatrix}, \\ \sigma_{\text{m},\Omega2}^{\text{HP}} &= (\sigma_{\text{bck}}+\sigma_{\text{act}})\cdot\begin{pmatrix}\cos^2\left(\frac{\pi}{3}\right) & \frac{1}{2}\sin\left(\frac{2\pi}{3}\right) \\ \frac{1}{2}\sin\left(\frac{2\pi}{3}\right) & \sin^2\left(\frac{\pi}{3}\right)\end{pmatrix}, \\ \sigma_{\text{m},\Omega3}^{\text{HP}} &= (\sigma_{\text{bck}}+\sigma_{\text{act}})\cdot\begin{pmatrix}\cos^2\left(\frac{\pi}{3}\right) & -\frac{1}{2}\sin\left(\frac{2\pi}{3}\right) \\ -\frac{1}{2}\sin\left(\frac{2\pi}{3}\right) & \sin^2\left(\frac{\pi}{3}\right)\end{pmatrix},\end{aligned} \tag{31}$$

for the respective regions Ω_1, Ω_2 and Ω_3 in the case of the hazard pattern (HP).

We consider three possible models for the time course of the PA stress component σ_{act}. The simplest case is the rectangular profile

$$\sigma_{\text{act}}^{\text{rec}}(t) = \begin{cases}\sigma_0 & \text{for } t_0 \le t \le t_{\text{act}} \\ 0 & \text{else}\end{cases}, \tag{32}$$

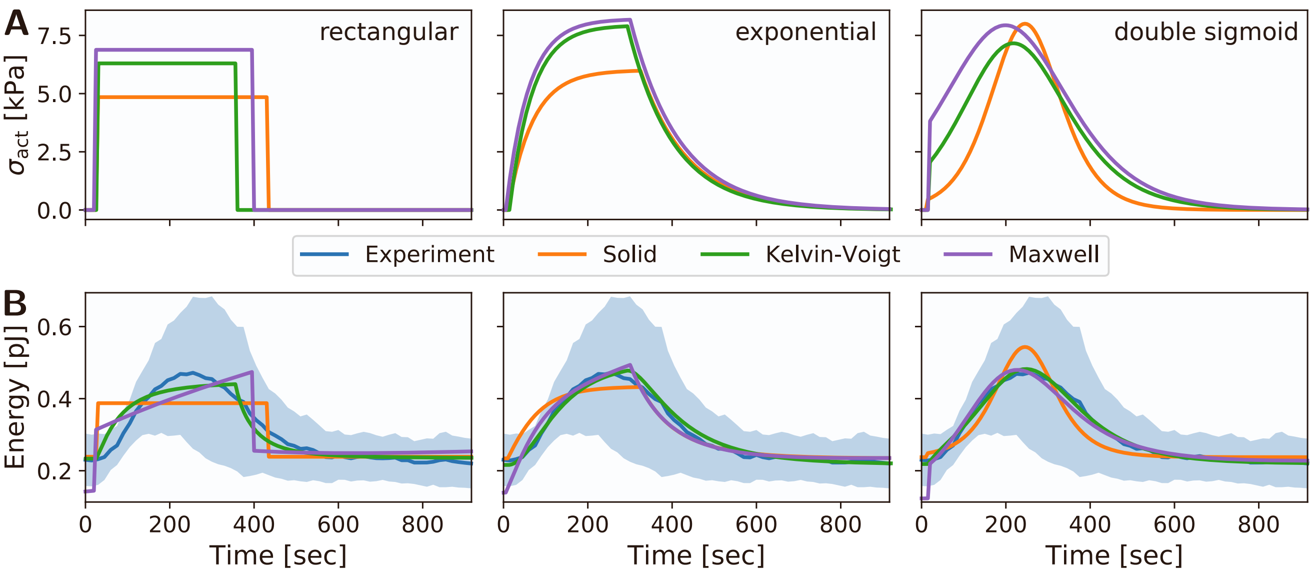

Figure 4: Photoactivation stress profile σ_{act} and the corresponding energy response for the case of a solid, Kelvin-Voigt and Maxwell model. (A) Photoactivation stress profiles σ_{act} used to reproduce the experimentally acquired cellular energy response: rectangular, exponential and double sigmoid profile from left to right. Different curves illustrate the optimized stress profiles for the three models. (B) Corresponding energy responses for the three different stress profiles and continuum models, illustrated on top of the experimental average. *Shaded regions* denote the standard deviation. A Kelvin-Voigt model with a double sigmoid stress profile fits best to the experimental curve.

with peak activation stress σ_0, PA time point t_0 and duration t_{act}. To account for a delayed response of the activation stress, we introduce the exponential profile

$$\sigma_{act}^{exp}(t) = \begin{cases} \sigma_0 \left(1 - \exp\left(-\frac{t-t_0}{\tau_{act}}\right)\right) & \text{for } t_0 \leq t \leq t_{act} \\ \sigma_0 \left(1 - \exp\left(-\frac{t_{act}-t_0}{\tau_{act}}\right)\right) \exp\left(-\frac{t-(t_0+t_{act})}{\tau_{rel}}\right) & \text{else} \end{cases}, \tag{33}$$

with stress activation and relaxation times τ_{act} and τ_{rel}. The third stress profile is a double sigmoid function [10]

$$\sigma_{act}^{sig}(t) = \frac{\sigma_0}{1 + \exp\left(-\frac{t-t_{act}}{\tau_{act}}\right)} \cdot \left(1 - \frac{1}{1 + \exp\left(-\frac{t-t_{rel}}{\tau_{rel}}\right)}\right), \tag{34}$$

with the centers of the activating and relaxing sigmoid t_{act} and t_{rel}. Here, the two time constants and the two time centroids allow us to combine a discontinuous jump at the ascending edge of the PA stress function and a flat stress plateau, as with the rectangular stress profile, with a damped activation and relaxation, as with the exponential stress profile. Thus the sigmoid activation can be considered to be the most general form of an activation profile.

These three different PA-stresses now can be combined with each of the four mechanical models introduced above to select the best model for further analysis. A purely viscous model can be excluded right from the beginning as it would not allow to keep a steady energy state, even in the absence of a PA-signal. In Fig. 4, we show the responses of the three other potential models, each time combined

Fixed parameter	Value
Young's modulus of the substrate E_s	4.47 kPa
Poisson's ration of the substrate ν_s	0.5
Substrate thickness h_s	50 µm
Lateral cell size L_c	50 µm
Young's modulus of the cell E_c	10 kPa
Viscosity of the cell η_c	100 kPa s
Poisson's ratio of the cell ν_c	0.5
Cell layer thickness h_c	1 µm

Table 1: Globally fixed cell and substrate parameters.

with one of the three potential PA-stresses after optimization against the experimental data. One sees that the best combination is the Kelvin-Voigt model combined with the double sigmoid. The rectangular PA-profile would introduce discontinous features in the strain energy response that are not present in the experiments. The exponential PA-profile would give an asymmetric response. Only the double sigmoid gives the smooth response observed experimentally. While the elastic model gives too steep curves, the Maxwell model needs different baselines before and after PA. The Kelvin-Voigt model gives near perfect fits.

8 Parametrization

In principle, one can minimize our theoretical predictions against our experimental readout (strain energy as a function of time) in regard to all model parameters. However, there are too many of them to get unique solutions and therefore we fix those parameters that are well established in the literature and only minimize for the ones that are specific to our experimental setup. Moreover the parameters of the substrate are known anyway. In Tab. 1, we list the fixed and known parameters. For the cell parameters, we use consensus values from the literature [1, 2, 6, 7]. In particular, cell stiffness is set to $E_\mathrm{c} = 10\,\mathrm{kPa}$, which is a typical value for strongly adherent cells. Setting cell viscosity to $\eta_\mathrm{c} = 100\,\mathrm{kPa}\cdot\mathrm{s}$ corresponds to a viscoelastic relaxation time of $\tau = 10\,\mathrm{s}$.

We next estimate the stiffnesses of substrate and adhesion layer. With $E_\mathrm{s} = 4.47\times10^3\,\mathrm{kPa}$, $h_\mathrm{s} = 50\,\mu\mathrm{m}$ and $L_\mathrm{c} \approx 50\,\mu\mathrm{m}$, we have for the substrate approximately $Y_\mathrm{s} \approx 3\times10^8\,{}^{N}\!/\mathrm{m}^3$. For the effective spring constant of adhesions, a standard value is $k_a = 2.5\,\mathrm{nN}/\mu\mathrm{m} = 2.5\,\mathrm{pN}/\mathrm{nm}$ and a typical dimension is $d = 1\ \mu m$. Thus we estimate $Y_\mathrm{a} \approx k_a/\mu m^2 \approx 2\times10^9\,{}^{N}\!/\mathrm{m}^3$. This suggests that the adhesion layer is the stiffer element and that the cells perceive mainly the stiffness of the substrate.

The two parameters that are fitted to the baseline before PA are localization length l_p and background stress σ_back. These quantities represent the main characteristics of adhesion and force generation and together determine the substrate strain stored in the elastic substrate. Tab. 2 documents our results for the four different patterns used in the main text.

For the photoactivation part, we fit five parameters: the peak value for the double sigmoid σ_0, and its

Fit parameter	Disc 500 μm	Disc 1000 μm	Disc 1500 μm	Hazard
Force localization length l_{p}	2.65 μm	3.75 μm	4.62 μm	2.97 μm
Contractile background stress σ_{back}	2.23 kPa	3.91 kPa	5.30 kPa	3.58 kPa

Table 2: Fit results for energy baseline.

Fit parameter	Disc 500 μm	Disc 1000 μm	Disc 1500 μm	Hazard
σ_0	1.2 kPa	1.8 kPa	1.8 kPa	0.8 kPa
σ_{max}	1.2 kPa	1.7 kPa	1.7 kPa	0.7 kPa
t_{act}	46 s	79 s	66 s	59 s
t_{rel}	291 s	416 s	343 s	335 s
τ_{act}	13 s	33 s	19 s	20 s
τ_{rel}	42 s	60 s	52 s	78 s

Table 3: Fit parameter for 100 ms PA-duration.

four time values. From this, one can calculate also the maximal stress σ_{max} achieved during PA. The corresponding results are given in Tab. 3. The results for the pulses are given in Tab. 4. The resulting strain energy curves and their interpretations are given in the main text.

9 Scaling considerations from analytical model

We now can use our analytical solution for the isotropic contractile disc in the limit of large disc size to rationalize our findings. We first note that a typical overall cell force is $F_{tot} = \mu N$. We therefore estimate for the background stress

$$\sigma_{\mathrm{back}} = \frac{F_{tot}}{2\pi r_0 h_c} \approx 10\ kPa \tag{35}$$

in very good agreement with the order of magnitude of our fit results. We also note that the background stress sets the order of magnitude for cell elasticity, which we here fix to $E_{\mathrm{c}} = 10\,\mathrm{kPa}$.

For the localization length, we estimate

$$l_p = \left(\frac{E_{\mathrm{c}} h_{\mathrm{c}}}{Y\left(1-\nu_{\mathrm{c}}^2\right)}\right)^{1/2} \approx 1\ \mu m \tag{36}$$

again in very good agreement with the order of magnitude of our fit results.

Finally for the strain energy we estimate

$$U_{\mathrm{s}} = \frac{\pi\left(\sigma_0 h_{\mathrm{c}}\right)^2}{2Y_{\mathrm{s}}}\frac{r_0}{l_{\mathrm{p}}} \approx 1\ pJ \tag{37}$$

again in very good agreement with the experimentally measured and fitted order of magnitude. Together, these estimates show that our theory is consistent and predictive.

PA duration	10 ms	20 ms	50 ms	100 ms	150 ms	200 ms
Fit parameter	Values					
			Disc			
σ_0	0.6 kPa	1.1 kPa	1.5 kPa	1.7 kPa	1.9 kPa	1.9 kPa
σ_{max}	0.6 kPa	1.1 kPa	1.4 kPa	1.6 kPa	1.9 kPa	1.8 kPa
t_{act}	72 s	71 s	89 s	105 s	96 s	105 s
t_{rel}	255 s	324 s	382 s	453 s	462 s	465 s
τ_{act}	10 s	21 s	30 s	33 s	49 s	48 s
τ_{rel}	34 s	35 s	48 s	53 s	39 s	67 s
			Hazard			
σ_0	0.9 kPa	1.0 kPa	0.9 kPa	0.9 kPa	0.9 kPa	0.9 kPa
σ_{max}	0.3 kPa	0.5 kPa	0.7 kPa	0.8 kPa	0.9 kPa	0.8 kPa
t_{act}	34 s	49 s	50 s	63 s	62 s	77 s
t_{rel}	130 s	186 s	267 s	278 s	350 s	275 s
τ_{act}	16 s	27 s	16 s	15 s	12 s	14 s
τ_{rel}	141 s	102 s	74 s	54 s	45 s	57 s

Table 4: Fit parameter for long opto protocol. Stresses rounded to one digit after comma.

10 Effect of actin organization on strain energy level in the model

Since in our model fits yield different l_p for hazard and disc pattern, respectively which influences the substrate strain energy $U_s \sim Y^2 \sim 1/l_p^4$ we choose a set of dummy parameters to purely study the influence of the adhesion geometry on the substrate strain energy. As in our example for the isotropic contractile disc we set $\sigma_{\text{back}} = 4$ kPa and $l_p = 4$ µm. This yields baseline strain energy values of $U_{\text{s}}^{\text{Hazard}} = 0.29$ pJ and $U_{\text{s}}^{\text{Disc}} = 0.24$ pJ. Since all cell parameters are identical we conclude that the strain energy is strongly influenced by the internal stress fiber organization. The length of the "effective" boundary is given by $B^{\text{Hazard}} = (\frac{3}{2}\pi + 3)r_0$. Since U_{s} is essentially proportional to the length along which the traction acts (marked in red for the hazard) we can compute the ratio of B and the circumference of a circle, which is the effective boundary for the circle pattern, and compare it to the ratio of the two strain energy values listed above. The first ratio yields a value of $B^{\text{Hazard}}/(2\pi r_0) = 1.23$ while the ratio of the strain energy gives $U_{\text{s}}^{\text{Hazard}}/U_{\text{s}}^{\text{Disc}} = 1.21$ such that this very minimal consideration could explain the observed strain energy difference.

However, it is not possible to follow the same argumentation when it comes to strain energy response upon photo activation. Additionally, the model alone is not sufficient to explain this observation since model parameters where fitted such that they resemble the experimentally measured strain energy response. Very generally, the less effective force generation on the hazard pattern could be the result of either more stressed stress fibers which have less force generation capacity or simply the result of differing stress fiber densities within the two conditions. Regarding the very similar baseline stress

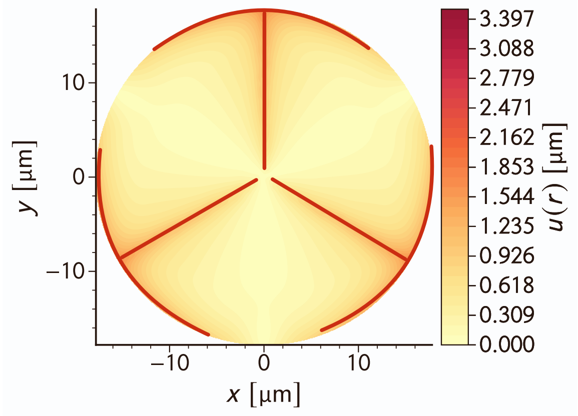

Figure 5

levels for hazard and disc those two explanations could be equivalent.

11 Example for python FEM-code for contractile disc

Our model was implemented in the FEM-framework FEniCS [9]. As an example of our code, here we document the calculation for the isotropic contractile disc.

```
from dolfin import *
import numpy as np
import sys
import logging
logging.basicConfig(level=logging.DEBUG)
logger = logging.getLogger("rothemain.rothe_utils")
logging.getLogger('UFL').setLevel(logging.WARNING)
logging.getLogger('FFC').setLevel(logging.WARNING)
set_log_active(False)
tol_x = DOLFIN_EPS
tol_y = DOLFIN_EPS
''' Sample script for the Finite Element Simulation of an isotropic contractile disc with elastic foundation.
    Script was written for the 2019 version of FEniCS and run inside a Docker container build from the 2019 image quay.io/fenicsproject/stable:"version"

    Copyright: Dennis Woerthmueller, Dimitri Probst
    Last modification: August 29, 2021
'''
# Strain
def eps(v):
    # Calculate the symmetric strain tensor.
    return sym(grad(v))

# Stress
def sigma(v, lmbda, mu):
    # Calculate the stress tensor based on constitutive relation for a linear elastic solid.
    return 2.0 * mu * eps(v) + lmbda * tr(eps(v)) * Identity(len(v))

# Active stress tensor
def active(sx, sy, sxy=0.0):
    # Define the tensor for the active stress contribution.
    return as_tensor([[sx, sxy], [sxy, sy]])
```

```

# Calculation of the penetration length on a thick substrate
def penetrationLength_thick_subs(Ec, hc):
    # Formulas taken from: Banerjee & Marchetti (2012): Contractile stresses in cohesive cell
    # layers on finite-thickness substrates
    ka = 2.5e-3          # Stiffness of focal adhesion bonds [N/m]
    L = 50e-6            # Cell length (1d), diameter (2d) [m]
    lc0 = 1e-6           # Length of sarcomeric subunit [m]
    hs = 50e-6           # Thickness of the substrate [m]
    nus = 0.5            # Poisson's ratio of the substrate
    Es = 4.47e3          # Elastic modulus of the substrate [N/m^2]
    heff = (1. / (hs * 2 * np.pi * (1 + nus)) + 1. / L)**-1
    Ya = ka / (L * lc0)
    Ys = (np.pi * Es) / heff
    Y = (1.0 / Ya + 1.0 / Ys)**(-1)
    #print Ya, Ys
    lp = np.sqrt(Ec * hc / Y)
    return lp, Ys / 1e12

# only relevant if symmetry if pattern is present and if PA is on full cell
def DirichletBoundary(x, on_boundary):
    # Define the diriclet boundary condition for center of the circular cell.
    return near(x[0], 0.0, tol_x) and near(x[1], 0.0, tol_y)

# strain energy
def calculateStrainEnergy(u, kN, Ys, F, V, assigner_V_to_F, mesh):
   # calculate strain energy of the cell according to formula defined in the theory supplement.
    ux = Function(F)
    uy = Function(F)
    u0 = Function(V)
    u0.assign(u)
    u0.vector()[:] *= u0.vector()
    # Split so that ux = ux**2, uy = uy**2
    assigner_V_to_F.assign([ux, uy], u0)
    # ux will hold |u|**2 = ux**2 + 1 * uy**2
    ux.vector().axpy(1, uy.vector())
    return assemble(0.5*kN**2/Ys*ux*dx(mesh))

# function which defines the simulation
def isotropic_contractile_disc():

    # Import a pre-created mesh (e.g. with gmsh)
    mesh = Mesh('circ_1000.xml')

    # set fixed cell parameters
    E3D = 10e3 # Elastic modulus of the cell in Pa
    eta3D = 100e3 # Viscous modulus of the cell  Pa*s
    h = 1e-6 # cell height in m
    sigma_back2D = 4e-03 # 2D stress N/m, conversion between 2D and 2D via sigma_2D = sigma_3D*h
    sigma0 = 5e-03 # 2D active stress in N/m

    # conversion to 2D constants for plane stress and thin layer approximation
    Eh = E3D * h # N / m = Pa * m
    etah = eta3D * h # Ns / m = Pa * m
    nu = 0.5
    lmbdaE = Eh * nu / ((1 - nu) * (1 + nu)) # in 3D given by: ((1 + nu) * (1 - 2 * nu))
    muE = Eh / (2 * (1 + nu))
    lmbdaEta = etah * nu / ((1 - nu) * (1 + nu)) # in 3D given by: ((1 + nu) * (1 - 2 * nu))
    muEta = etah / (2 * (1 + nu))
    _, Ys = penetrationLength_thick_subs(E3D, h) # Unit [m]. Here, only Ys is calculated!
    lp  = 4*1e-6 # force penetration length in m; in our approach a fit parameter of strain energy baseline fit
    kN = Constant((lmbdaE + 2 * muE) / (lp * 1e6)**2) # Spring stiffness density kN in N / m / um**2 to get u (displacement field) in um

    # Define Time Stepping
```

```
dt = Constant(15) # Time constant in s, definde in UFL (unified form language) which is built on top of the python language
T = 1005 # Total simulation time in s, make sure that T mod dt= 0 otherwise last time step is missing
lag_time = 3 * etah / Eh  # Natural time scale of the system which describes the time the cell needs to arrive in its 'ground state'.
act_times = np.array([300,T+15]) + lag_time # Time points of photo activation stress

# Define function space and basis functions
V = VectorFunctionSpace(mesh, "CG", 2) # continuous Galerkin of degree 2 (Lagrange polynomials)
u = TrialFunction(V)
v = TestFunction(V)

# Define boundary condition
u0 = Constant((0.0, 0.0)) # zero displacement in symmetry center of the disc
bc = DirichletBC(V, u0, DirichletBoundary)

# Define variational form in Dolfin UFL syntax with a backward euler time discretization scheme
a = inner(sigma(u, lmbdaEta, muEta), sym(grad(v)))*dx + dt*inner(sigma(u, lmbdaE, muE), sym(grad(v)))*dx + dt * kN * inner(u, v) * dx
u = Function(V)
uinit = Constant((0.0, 0.0))
uold = interpolate(uinit, V)
uold.assign(u)

#  Define Elements and Function Space for resulting Tensors
F = FunctionSpace(mesh, 'CG', 2)
assigner_V_to_F = FunctionAssigner([F, F], V)
dFE = FiniteElement("DG", mesh.ufl_cell(), 0)
tFE = TensorElement(dFE)
W = FunctionSpace(mesh, tFE)
K = FunctionSpace(mesh, dFE)
stress = Function(W, name='Stress')
disp = Function(V, name='Displacement')

# Determine the save options and save resulting fields to output.xdmf to view with ParaView
xdmf_file= XDMFFile("simulation_result.xdmf")
xdmf_file.parameters["flush_output"] = True
xdmf_file.parameters["functions_share_mesh"] = True
save = True

# Initialize lists to save simulation results
all_times = []
all_energies = []

# Run simulation
t = 0 * dt # start time
lag_counter = 0 # ounts the number of time needed for lag time
act_flag = False # True, if activated
sigma_act = 0.0 # initial photo activation stress

# in the example we use an exponetial shaped activation profile with the following free time parameters:
tau_stress_act = 30 # time scale for activation
act_duration = 300 # duration photo activation
tau_stress_rel = 40 # time scale for relaxation

# main simulation loop, time evolution. Solve system for each time step.
while t(0.0) <= T + lag_time:
    print(t(0.0))
    if t(0.0) < lag_time: # count the number of time steps necessary for lag time
        lag_counter += 1
    if near(t(0.0), act_times[0]) and act_flag == False: # set act_flag to true if first activation time point is reached
        print("GOT ACTIVATED")
        act_flag = True
```

```
        if act_flag == True:  # calculate the active stress contribution which is != 0
            act_time = act_times[0]
            newT = t(0.0) - act_time # times in activation function are measure relative to activation time point
            if newT <= act_duration: # acitvation
                sigma_act = sigma0 * (1 - np.exp(-newT/ tau_stress_act))
            else: # relaxation
                sigma_act = sigma0 * (1 - np.exp(-act_duration / tau_stress_act)) * np.exp(-(newT-act_duration)/tau_stress_rel)
        if sigma_act < 1e-08:
            sigma_act = 0.0

        # Right side of variational form
        L = inner(sigma(uold, lmbdaEta, muEta),sym(grad(v)))*dx - \
            dt * inner(active(sigma_back2D+sigma_act, sigma_back2D+sigma_act), sym(grad(v))) * dx

        # Solve problem with boundary conditions bc
        solve(a == L, u, bc)
        uold.assign(u) # assign solution to uold to use in next iteration time step
        total_energy = calculateStrainEnergy(u, kN, Ys, F, V, assigner_V_to_F, mesh)   # Unit pJ

        # store data
        all_times.append(t(0.0))
        all_energies.append(total_energy)

        # calculate total stress/strain tensors
        eps = sym(grad(u))
        sig = active(sigma_back2D+sigma_act, sigma_back2D+sigma_act) + Eh/(1+nu)*eps + nu*Eh/(1-nu**2)*tr(eps)*Identity(2)
        stress.assign(project(sig, W))
        disp.assign(u)

        # save tensors at each time step to xdmf-file
        if save:
            xdmf_file.write(disp, t(0.0))
            xdmf_file.write(stress, t(0.0))
        # update time step
        t += dt

    return None
# Main function
if __name__ == "__main__":

    isotropic_contractile_disc() # simulate the photo activated isotropic contractile disc
```

References

[1] C.M. Edwards and U.S. Schwarz. Force localization in contracting cell layers. *Physical Review Letters*, 107(12):128101, 2011.

[2] S. Banerjee and M.C. Marchetti. Contractile stresses in cohesive cell layers on finite-thickness substrates. *Physical Review Letters*, 109(10):108101, 2012.

[3] Aaron F Mertz, Shiladitya Banerjee, Yonglu Che, Guy K German, Ye Xu, Callen Hyland, M Cristina Marchetti, Valerie Horsley, Eric R Dufresne, et al. Scaling of traction forces with the size of cohesive cell colonies. *Physical Review Letters*, 108(19):198101, 2012.

[4] Shiladitya Banerjee and M Cristina Marchetti. Controlling cell–matrix traction forces by extracellular geometry. *New Journal of Physics*, 15(3):035015, 2013.

[5] P.W. Oakes, S. Banerjee, M.C. Marchetti, and M.L. Gardel. Geometry regulates traction stresses in adherent cells. *Biophysical Journal*, 107(4):825–833, 2014.

[6] Medhavi Vishwakarma, Jacopo Di Russo, Dimitri Probst, Ulrich S Schwarz, Tamal Das, and Joachim P Spatz. Mechanical interactions among followers determine the emergence of leaders in migrating epithelial cell collectives. *Nature Communications*, 9(1):1–12, 2018.

[7] Jana Hanke, Dimitri Probst, Assaf Zemel, Ulrich S Schwarz, and Sarah Köster. Dynamics of force generation by spreading platelets. *Soft Matter*, 14(31):6571–6581, 2018.

[8] Josephine Solowiej-Wedderburn and Carina M Dunlop. Sticking around: Cell adhesion patterning for energy minimization and substrate mechanosensing. *Biophysical Journal*, 121(9):1777–1786, 2022.

[9] M.S. Alnaes, J. Blechta, J. Hake, J. Johansson, B. Kehlet, A. Logg, C. Richardson, J. Ring, M.E. Rognes, and G.N. Wells. The FEniCS project version 1.5. *Archive of Numerical Software*, 3(100):9–23, 2015.

[10] Patrick W Oakes, Elizabeth Wagner, Christoph A Brand, Dimitri Probst, Marco Linke, Ulrich S Schwarz, Michael Glotzer, and Margaret L Gardel. Optogenetic control of rhoa reveals zyxin-mediated elasticity of stress fibres. *Nature Communications*, 8:15817, 2017.
